# Serological evidence of tick-borne encephalitis virus infection in moose and deer in Finland: sentinels for virus circulation

**DOI:** 10.1186/s13071-016-1335-6

**Published:** 2016-01-29

**Authors:** Elina Tonteri, Pikka Jokelainen, Juho Matala, Jyrki Pusenius, Olli Vapalahti

**Affiliations:** Department of Virology, University of Helsinki, Faculty of Medicine, Helsinki, Finland; Department of Veterinary Biosciences, University of Helsinki, Faculty of Veterinary Medicine, Helsinki, Finland; Department of Food Hygiene and Environmental Health, University of Helsinki, Faculty of Veterinary Medicine, Helsinki, Finland; Department of Basic Veterinary Sciences and Population Medicine, Estonian University of Life Sciences, Tartu, Estonia; Natural Resources Institute Finland (Luke), Management and Production of Renewable Resources, Joensuu, Finland; Department of Virology and Immunology, Hospital district of Helsinki and Uusimaa (HUSLAB), Helsinki, Finland

**Keywords:** Moose, Roe deer, Serology, Tick-borne encephalitis, White-tailed deer, Zoonosis

## Abstract

**Background:**

The incidence of tick-borne encephalitis (TBE) in humans has increased in Finland, and the disease has emerged in new foci. These foci have been investigated to determine the circulating virus subtype, the tick host species and the ecological parameters, but countrywide epidemiological information on the distribution of TBEV has been limited.

**Methods:**

In this study, we screened sera from hunter-harvested wild cervids for the presence of antibodies against tick-borne encephalitis virus (TBEV) with a hemagglutination inhibition test. The positive results were confirmed by a neutralisation assay.

**Results:**

Nine (0.74 %) of 1213 moose, one (0.74 %) of 135 white-tailed deer, and none of the 17 roe deer were found seropositive for TBEV. A close geographical congruence between seropositive cervids and recently reported human TBE cases was observed: nine of the ten seropositive animals were from known endemic areas.

**Conclusions:**

Our results confirm the local circulation of TBEV in several known endemic areas. One seropositive moose had been shot in an area where human TBE cases have not been reported, suggesting a possible new focus. Moose appear to be a useful sentinel animal for the presence of TBEV in the taiga region.

## Background

Tick-borne encephalitis virus (TBEV) is a zoonotic arbovirus. In 1991–2010, it caused up to 12,733 annual reported human cases of tick-borne encephalitis (TBE) in Europe [1]. There are three known genetic subtypes of the virus: European, Siberian and Far-Eastern [2]. The subtypes differ in their endemic regions and ecological preferences, and possibly in their pathogenicity [3].

The virus is maintained in an enzootic cycle of ticks, which are vectors and hosts for the virus, and their vertebrate hosts. Non-viremic transmission (NVT) of TBEV between ticks co-feeding on small vertebrates is considered important for the maintenance of the virus [4]. Due to the complex ecology of TBEV, its geographical distribution is multifocal [5].

Large mammals are secondary hosts for TBEV as they support the tick populations by providing blood meals. The NVT competence of large mammals is controversial and largely unknown: goats are not NVT-competent, whereas the NVT potential of roe deer (*Capreolus capreolus*), which can be heavily infested by co-feeding larvae and nymphs, has not been studied [6–10]. Nevertheless, roe deer and red deer (*Cervus elaphus*) appear to be key hosts for ticks and thereby important for TBEV maintenance [8–10]. In Sweden, the increase in TBE incidence has been associated with changes in cervid populations, especially roe deer populations [11].

TBE is usually subclinical in middle-sized and large mammals [12]. However, in Sweden, a moose (*Alces alces*) calf with severe behavioral abnormalities and subsequently diagnosed encephalitis was found to be TBEV-positive [13].

Finland is at the northern boundary of the current distribution area of TBEV [14]. Two subtypes of the virus, European and Siberian, as well as the two main host tick species, *Ixodes ricinus* and *I.persulcatus*, have been described [15]*.* Between 2007 and 2014, a total of 273 human TBE cases were reported in Finland [16, 17]. The cases occurred mainly in known endemic areas, where human TBE cases have been diagnosed for decades, and where TBEV antibodies were detected in cattle already in the 1960s [16, 18]. The incidence of human TBE has increased, and the disease has emerged in new foci [16]. Bank vole (*Myodes glareolus*) appears to be the dominant small mammal in the studied TBEV foci in Finland in the boreal taiga region [14, 19]. Large and middle-sized mammalian species supporting the local maintenance of TBEV have not been investigated.

There are three wild cervid species with substantial populations in Finland. Since the1970's, the moose population has comprised 60,000 to 200,000 individuals that are evenly distributed across the country [20, 21]. The white-tailed deer (*Odocoileus virginianus*) population has 40,000 to 50,000 individuals, that inhabit mainly south-western Finland [22]. The roe deer population of 10,000 to 20,000 individuals is distributed sparsely across the country, with the highest numbers in the south-west [23].

In this study, we screened sera of these three wild cervid species for TBEV-specific antibodies to estimate the seroprevalence and to determine the geographical distribution of TBEV in Finland. We discuss the suitability of moose as a sentinel animal for the presence of TBEV and as an indicator for local risk of human TBE infections.

## Methods

### Ethics statement

No animals were killed for the purpose of this study. The animals were sampled post mortem, after they had been legally killed by hunters. The samples were stored, coded, and all data were treated confidentially.

### Study design and sampling

The serum bank was collected during the hunting season of 2008–2009, primarily for a nationwide cross-sectional serological study on *Toxoplasma gondii* [24]. The sample is a convenience sample, and the sampling was not targeted for TBEV foci. Altogether 2917 sampling packages were distributed to the game management districts, which subsequently distributed them to the hunters. Each package included two plastic VACUETTE® Serum Clot Activator blood sample tubes (Greiner Bio-One GmbH, Kremsmünster, Austria) and a questionnaire covering the game management district, and species, sex and age group of the animal sampled.

All the animals included in the study were legally hunted for human consumption. The voluntarily participating hunters collected the samples from the animals and filled out the questionnaire for each animal. The participation rate was 47 %. Samples were sent by mail and arrived at the laboratory within 4 days of sampling, between September 15th 2008 and February 2nd 2009. Upon arrival, the samples were coded, and sera were separated by centrifugation, divided into aliquots and stored at - 20 °C until analyzed. Only the code number of each sample was known by the persons performing the tests.

A total of 1371 cervid samples were included in this study. The samples from 1213 moose originated from all 15 game management districts. The deer samples, from 135 white-tailed deer and 17 roe deer, had been collected only from the south-western districts where these cervids are the most numerous. The species of six samples was unspecified.

### Serological methods

For the serological analysis for the presence of anti-TBEV antibodies, we used an in-house hemagglutination inhibition (HI) test [25] using the following two-fold dilutions: 1:10, 1:20, 1:40, 1:80, 1:160, 1:320, 1:640. Titers were determined according to these dilutions, however, in cases where the last dilution showed partial inhibition of hemagglutination, the titer was determined as the value between the last clear dilution and the borderline dilution and is presented in Table 1 by showing both the lower and higher dilutions. Positive results were further confirmed by a rapid focus-forming inhibition test for neutralizing antibodies using Swedish European subtype strain 93–783 [25]. The test was performed in dilutions 1:5 and 1:20. The samples that tested positive in the HI test and positive or borderline in the neutralisation test were defined as seropositive. In addition, the samples were screened with in-house HI tests for the presence of antibodies against two other flaviviruses that could cause cross-reactive antibody responses: West Nile virus (WNV), which has not been found in Finland, and Lammivirus (LAMV) which has been found in mosquitoes in Finland [26].

**Table 1:** Basic data and serological results of the wild cervids hunted in 2008–2009 in Finland that tested seropositive in the screening for antibodies against tick-borne encephalitis virus by hemagglutination inhibition test

Individual	Species	Age	Sex	Disrtict^a^	HI	Neutralisation			HI	HI
					TBEV	TBEV			WNV	LAMV
						NT 5	NT 20	NT result		
1.	moose	adult	M	8	640	20/20	20/20	neg	neg	20–40
2.	moose	adult	M	1	160–320	0/20	6/20	pos	<10	neg
3.	moose	calf	M	8	160–320	9/20	17/20	pos	neg	neg
4.	moose	adult	M	5	80–160	0/20	20/20	pos	neg	neg
5.	moose	nd.	F	8	80	4/20	15/20	pos	neg	neg
6.	moose	adult	M	1	80	0/20	10/20	pos	neg	neg
7.	moose	adult	M	13	80	20/20	20/20	neg	neg	neg
8.	moose	adult	M	3	80	2/20	17/20	pos	neg	neg
9.	moose	calf	M	14	40–80	10/20	20/20	pos/neg	neg	neg
10.	moose	adult	M	7	40	0/20	13/20	pos	neg	20–40
11.	moose	calf	F	13	40	20/20	20/20	neg	neg	neg
12.	moose	adult	M	14	<40	20/20	20/20	neg	neg	neg
13.	moose	adult	F	10	20–40	20/20	20/20	neg	neg	neg
14.	moose	calf	M	7	20	20/20	20/20	neg	neg	neg
15.	deer ^b^	adult	M	1	20	12/20	20/20	pos/neg	neg	neg
16.	moose	calf	M	4	20	20/20	20/20	neg	neg	neg
17.	moose	calf	F	3	20	16/20	20/20	neg	neg	10
18.	moose	adult	F	12	20	18/20	20/20	neg	neg	neg
19.	moose	adult	M	5	<20	20/20	20/20	neg	neg	neg
20.	moose	adult	F	1	10–20	20/20	20/20	neg	neg	neg
21.	moose	adult	M	8	10–20	14/20	20/20	pos/neg	10	10–20
22.	moose	adult	M	1	10–20	20/20	20/20	neg	neg	neg
23.	moose	adult	M	7	10–20	17/20	20/20	neg	neg	neg
24.	moose	calf	M	3	10–20	20/20	20/20	neg	neg	neg
25.	moose	adult	F	3	10–20	20/20	20/20	neg	neg	neg
26.	moose	adult	M	1	10–20	20/20	20/20	neg	neg	neg
27.	moose	adult	F	8	10–20	16/20	20/20	neg	neg	neg

^a^Districts presented in Fig. 1. The game administrative districts are described according to The Finnish Wildlife Agency

^b^White-tailed deer

*NT 5* neutralisation test, dilution 1:5

*NT 20*, neutralisation test, dilution 1:20

*HI* hemagglutination inhibition test

*TBEV* tick-borne encephalitis virus

*WNV* West-Nile virus

*LAMV* Lammi virus

### Statistical analyses

Two-by-two tables and test statistics were used for preliminary comparisons [27]. Logistical regression analyses with Stata 11.0 (StataCorp, College Station, Texas, US) were used to evaluate effects of available explanatory variables (game management district, sex, species, age group) on the outcome variable, TBEV-seropositivity.

## Results

Altogether 28 samples tested positive for anti-TBEV antibodies with the HI test (Table 1), and titers ranged from 15 to 640. These sera were further subject to confirmatory analysis by the TBEV neutralisation test. Ten samples showed positive (<10/20) or borderline result with dilutions 1:5 (NT 5) or 1:20 (NT 20) and were thus defined as seropositive (Table 1). The samples showing TBEV-specific antibodies by the neutralisation test had higher HI-titers than those samples that tested positive only on the HI test. The seropositive animals consisted of five adult male moose, two adult female moose, two young male moose, and one adult male white-tailed deer.

**Fig. 1:**
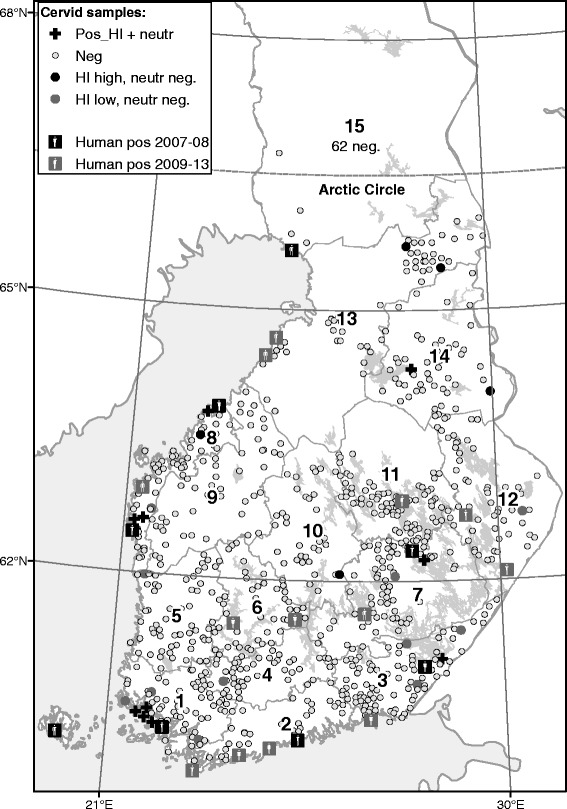
Geographical distribution of the hunting locations of the tested wild cervids, the animals defined as seropositive for antibodies against tick-borne encephalitis virus, and the cases of human tick-borne encephalitis in Finland. Data on hunting location were available for 79.2 % of the animals. The numbers 1–15 refer to the game management districts

The ten seropositive animals were from six different game management districts, and their geographical distribution appeared to correlate closely with the known human infection sites: nine seropositive animals originated from areas where human cases have been described (Fig. 1). The seroprevalences in moose and white-tailed deer were 0.74 %, and the seroprevalences did not differ significantly between the species, sexes and age groups (Table 2). Logistic regression analyses revealed no multivariable association.

**Table 2:** Prevalence of antibodies against tick-borne encephalitis virus in wild cervids hunted in 2008–2009 in Finland

	n^a^	n positive	Seroprevalence (%)	95 % confidence interval (Mid-P Exact)
Moose				
Male	672	7	1.04	0.46–2.05
Female	512	2	0.39	0.07–1.29
Adult	729	7	0.96	0.42–1.89
Calf	453	2	0.44	0.07–1.45
All	1213	9	0.74	0.36–1.36
White-tailed deer				
Male	77	1	1.30	0.07–6.24
Female	57	0	0.00	0.00–5.12
Adult	71	1	1.41	0.07–6.75
Calf	64	0	0.00	0.00–4.57
All	135	1	0.74	0.04–3.60
Roe deer				
Male	11	0	0.00	0.00–23.84
Female	6	0	0.00	0.00–39.30
Adult	14	0	0.00	0.00–19.26
Calf	3	0	0.00	0.00–63.16
All	17	0	0.00	0.00–16.16
All	1371	10	0.73	0.37–1.30

^a^Background information was unavailable for some of the animals investigated

None of the samples tested positive for WNV. Three of four samples that were weakly positive for LAMV, showed lower titers for this virus than for TBEV in the hemagglutination test (Table 1). One of the LAMV-positive samples showed a rather high hemagglutination titer, but this sample was still also positive for TBEV according to the TBEV neutralisation test (Table 1).

## Discussion

Presence of anti-TBEV-antibodies in moose has been previously investigated apparently only in Sweden in 1962 and more recently in Norway [13, 28]. In the Swedish study, the seroprevalence among 75 individuals was 44 %, which is almost sixty times higher than the Finnish estimate obtained in this study. The neutralisation test was used in both studies, but the results are not comparable because most of the animals included in the Swedish study had been shot in a known endemic area, whereas this study covered the whole of Finland except for the highly endemic Åland islands. In the Norwegian study, which used a commercial ELISA method, the prevalence was 52 %, but the sample size (*N* = 27) was small and the geographical range limited.

In this study, we used an in-house HI test as the primary screening test. Because flaviviruses cause cross-reactions in serological analyses, the positive results were confirmed by a labour-intensive, but more specific neutralisation test. To exclude some known cross-reactive flaviviruses, we tested the TBEV-positive samples (in the HI test) also for West Nile virus (WNV) and Lammivirus (LAMV). Low titers of LAMV-specific antibodies were detected in four animals in Southern and Central Finland, while there was no indication of WNV having been introduced in the cervid populations.

Finding serological evidence of TBEV infection in cervids indicates previous TBEV infection. A systemic infection is critical for the development of the specific antibody response. Non-viremic transmission (NVT) in rodents between co-feeding ticks is considered important for the maintenance of the virus and it does not require systemic infection [29, 30]. According to our unpublished observations and the earlier results by Svedmyr et al. [13], moose can be heavily infested by *Ixodes* ticks. The role of moose as secondary hosts for TBEV in the taiga region and their potential for NVT during tick co-feeding have not been investigated, but they are likely to have a role in amplifying the tick populations and potentially in introducing infected ticks to new areas.

In Central and Southern Europe as well as in large parts of Sweden, the roe deer is a key host for ticks, and a good indicator for the occurrence of human TBEV infections [8, 10, 11, 31–34]. The available estimates of TBEV seroprevalence in roe deer and red deer from other European countries range from 2.4 to 40 %, with marked local variation [31, 32, 35–38]. Due to the low number of roe deer samples in our study, the role of roe deer in the spread or circulation of TBEV in Finland could not be evaluated.

In Finland, moose is the most abundant and most widespread wild cervid, while the largest populations and the effective dispersal of roe deer and white-tailed deer are mainly limited to south-west Finland due to harsh winter conditions and thick snow cover in other parts of the country. However, small populations of roe deer exist in Lapland in shore and riverbank areas that have less snow [39, 40]. In Sweden, the geographic expansion of *I. ricinus* has followed the increase and dispersal of roe deer [11]. In Finland, the dispersal of white-tailed deer is not as effective and widespread as that of roe deer [41]. Interestingly, according to unpublished data of Finnish game authorities and the incidence and geographical distribution of human TBE [16], the emergence of new human cases seemed to coincide with the spread and increase of roe deer and white-tailed deer populations at the turn of the millenium in Finland. On the other hand, the same areas have had large moose populations and there are several other species that may also support the tick populations [42–44]. Most of the studies available have focused on single secondary host species. Sampling of all cervid species in known endemic areas might elucidate their roles in the spread and circulation of TBEV.

Collecting samples from cervids in co-operation with hunters proved successful and useful for evaluating the epidemiology of zoonotic diseases ([24] and this study). To analyse the role of moose for the local epidemiology and for the monitoring of TBEV locally and countrywide, repeated hunter-harvested sampling from cervids could be used alongside the continuous monitoring of human TBE. Good sample sizes are achievable, and sampling animals that were killed for another reason is ethically sound.

There were more males than females among the sampled animals, and male cervids dominate also in the annual hunting statistics in Finland [21, 45]. Eight of the ten seropositive animals were male. Male moose and young individuals migrate across a wider range and might therefore have more possibilities to encounter an infected tick and also to carry ticks to new locations [46]. In roe deer, higher TBEV-antibody prevalence has been seen in males even after the mobility is excluded [32]. In this study, the positive results were detected mainly in areas, which are known to be endemic for TBEV.

Among the animals included in this study, 37.9 % were calves (less than 1 year old), which is a smaller proportion than in the game statistics of Finland [21]. All animals included in the study had experienced at least one tick-feeding season, but the exact ages of the adult animals were not determined. Assuming that the antibodies persist, it cannot be estimated when the infections were acquired. Comparing the antibody prevalence in old individuals with the prevalence in young individuals could be used to determine whether the incidence of infections is increasing in the area. In this study, the prevalences were similar, suggesting recent introduction, or increasing infection pressure.

The only previous countrywide serological survey on the distribution and prevalence of TBEV infection in Finland was done in 1960s by screening TBEV antibodies in cattle serum samples [18]. The seropositive cattle were from areas where human cases have been diagnosed for decades. In this study, several seropositive wild cervids were shown to inhabit the same areas.

During the two previous tick-feeding seasons before the collecting of the cervid samples, human cases had been reported in eight areas in Finland (Fig. 1). In this study, seropositive cervids were detected in six of the eight areas. Finding no seropositive cervids in two of the eight areas may be explained by sampling bias: for example, the Helsinki focus is an island with no hunting activity. Finding no seropositives around the foci supports the previous observations that distribution of TBEV is highly focal.

One seropositive moose male calf (individual 9, Table 1) was from Kainuu, an area with no reports of human TBE cases. As a calf under 1 year of age follows a cow, and cows with calves usually do not do dispersal migrations, it is most likely that this individual had encountered the virus locally. This result could thus suggest a possible new TBEV focus, which calls for further monitoring of the area for TBEV circulation and potential human cases.

In Finland, the human TBE cases have emerged mainly nearby water – in the archipelago, coastal regions, and near big lakes [16]. This distribution of human TBE cases may be due to the high density of summer cottages by water as well as long exposure times during the summer holidays, which overlap with the tick feeding season. Other explanations for the proximity of human TBE cases to water include the ecological factors that cause the highly focal distribution of TBEV in favourable microclimatic conditions. Summer habitats of cervids are often also nearby water [47, 48] and moose are known to use water to cool off [49]. Furthermore, dispersal of roe deer has followed the seashores, rivers, and lakes [40]. These habitat preferences might enhance the potential role of cervids in maintaining tick populations that enable TBEV circulation.

During recent years, human TBE cases have been reported in new areas in Finland, and the strictly focal pattern may be changing towards a coalescing distribution [14–16, 50]. The results of this study suggest that moose could serve as sentinels and indicators for risk for human TBEV infections in the taiga region where deer, commonly used as sentinels for TBEV risk for humans, are not widespread. Surveying anti-TBEV antibodies in wild cervids could be a useful tool for monitoring this zoonosis.

## Conclusions

Anti TBEV-antibodies were detected in ten free-ranging cervids in Finland. The infections were presumably autochthonous, naturally acquired by the cervids from their local natural environment. Nine out of ten seropositive animals had been hunted in areas where human TBE cases have occurred, confirming the local circulation of the virus in the foci. Our study also found one possible new focus, as one seropositive moose male calf was from an area with no reports of human TBE cases. Our observation of the geographical congruence between the human cases and seropositive moose suggest that moose could serve as sentinels and indicators for TBEV risk to humans.

## Abbreviations

HI: hemagglutination inhibition test
LAMV: Lammi virus
NVT: non-viremic transmission
TBE: tick-borne encephalitis
TBEV: tick-borne encephalitis virus
WNV: West Nile virus
